# High fetal hemoglobin level is associated with increased risk of cerebral vasculopathy in children with sickle cell disease in Mayotte

**DOI:** 10.1186/s12887-020-02187-6

**Published:** 2020-06-20

**Authors:** Abdourahim Chamouine, Thoueiba Saandi, Mathias Muszlak, Juliette Larmaraud, Laurent Lambrecht, Jean Poisson, Julien Balicchi, Serge Pissard, Narcisse Elenga

**Affiliations:** 1 Pediatric Unit, Mamoudzou General Hospital, 1, Rue de l’Hopital, BP 4, 97600 Mamoudzou, Mayotte France; 2 APHP, GHU H Mondor, departement de genetique, INSERM-IMRB U955eq2/GREx, 51 Avenue du Maréchal de Lattre de Tassigny, 94010 Créteil Cedex, France; 3 Pediatric Medicine and Surgery, Cayenne General Hospital, Cayenne, French Guiana France

**Keywords:** Sickle cell disease, High hemoglobin level, Cerebral vasculopathy, Children, Single nucleotide polymorphism, Mayotte

## Abstract

**Background:**

Understanding the genetics underlying the heritable subphenotypes of sickle cell anemia, specific to each population, would be prognostically useful and could inform personalized therapeutics.The objective of this study was to describe the genetic modulators of sickle cell disease in a cohort of pediatric patients followed up in Mayotte.

**Methods:**

This retrospective cohort study analyzed clinical and biological data, collected between January1^st^2007 and December 31^st^2017, in children younger than 18 years.

**Results:**

We included 185 children with 72% SS, 16% Sβ0-thalassemia and 12% Sβ + thalassemia. The average age was 9.5 years; 10% of patients were lost to follow up. The Bantu haplotype was associated with an increase in hospitalizations and transfusions. The alpha-thalassemic mutation was associated with a decrease of hemolysis biological parameters (anemia, reticulocytes), and a decrease of cerebral vasculopathy. The Single Nucleotide Polymorphisms BCL11A rs4671393, BCL11A rs11886868, BCL11A rs1427407 and HMIP rs9399137 were associated with the group of children with HbF > 10%. Patients with HbF > 10% presented a significant risk of early onset of cerebral vasculopathy.

**Conclusions:**

The most remarkable result of our study was the association of SNPs with clinically relevant phenotypic groups. BCL11A rs4671393, BCL11A rs11886868, BCL11A rs1427407 and HMIP rs9399137 were correlated with HbF > 10%, a group that has a higher risk of cerebral vasculopathy and should be oriented towards the hemolytic sub-phenotype.

## Background

Sickle cell disease (SCD) refers to a group of autosomal recessive genetic disorders characterized by the synthesis of an abnormal hemoglobin: sickle hemoglobin S (*β*^*s*^, HbS), results from the substitution of a single amino acid (Glu → Val) at the sixth position of β-chain of normal hemoglobin (HbA) molecule [1, 2]. This single-point mutation leads to the polymerization of the HbS molecule and red cell sickling under deoxygenated conditions. Homozygous SS (sickle cell anemia or SCA) is usually considered the most severe form of SCD. Compound heterozygotes, in whom HbS is combined with a different mutation in the second β-globin gene, such as HbC, D, O^Arab^ or β-thalassemia (where β-globin synthesis is reduced) can also be affected, with variable phenotypes. SCD is characterized by abnormally shaped, adhesive red blood cells (RBCs) that interact with white blood cells (WBCs) and the endothelium, leading to chronic hemolysis, vasculopathy and a prothrombotic state [1]. These processes can result in severe complications including chronic pain, downstream-organ dysfunction, stroke, life-long suffering, poor quality of life and early mortality.

The clinical variability of SCD requires searching for factors responsible for its severity, in order to establish a clinical classification according to severity. This classification is useful for optimizing management, and adjusting the follow-up as closely as possible to the real risk presented by each patient. Thus, understanding the genetics underlying the heritable subphenotypes of SCD, specific to each population, would be prognostically useful and could inform customized therapeutics. Numerous studies have been devoted to genetic modulating factors of SCD [3–6]. Fetal hemoglobin (HbF) is the major genetic modulator of the hematologic and clinical features of SCD [7].

Coinheritance of alpha thalassemia trait and SCD is known to decrease the SCD severity. Indeed, alphathalassemia modulates SCD by reducing the intracellular concentration of HbS, which in turn reduces the HbS-polymer induced cellular damage. By the basis of this mechanism, there will be a reduction in hemolysis, stroke, silent infarction, transcranial doppler (TCD) velocity, and acute chest syndrome [8]. The β^S^-mutation is found on five haplotypes, that are named according to their putative geographic origins: Benin, Bantu (Central African), Cameroon, Senegal and Arab-Indian [9]. Many authors have tried to correlate the clinical severity of SCD with the beta globin haplotypes (βS). Despite some contradictory results, it is generally recognized that the Senegal and Arabic-Indian haplotypes are associated with fewer complications because of higher residual HbF levels. However, many studies were conducted in populations with only one or two over-represented βS haplotypes [10, 11].

Other genetic polymorphisms with an established influence on the SCD phenotype have been identified, including, HbF modifiers (XmnI, BCL11A, and HBS1L-MYB polymorphisms), uridine-diphosphoglucuronate glucuronosyltransferase (UGT1A1) promoter polymorphisms, and Glucose-6-phosphate dehydrogenase (G6PD) deficiency [12–14].

Additional candidate genes associated with subphenotypes of SCD have been described [15]. Clinical manifestations of SCD are generally not apparent until the switch from HbF to HbS occurs after the 3rd month of life [15]. This beneficial effect of HbF has been noted in patients who are compound heterozygotes for HbS and for hereditary persistence of fetal hemoglobin, or for other genetic variants of SCA with elevated HbF levels. Fetal hemoglobin genes regulation impacts the level of HbF and its distribution among sickle erythrocytes is highly variable [16, 17]. Little is known on genetic modifiers of SCD severity in Mayotte [18]. This article aims to describe the genetic modulators of SCD in a cohort of pediatric patients followed up in Mayotte between 2007 and 2017.

## Methods

### Study location

**Fig. 1:**
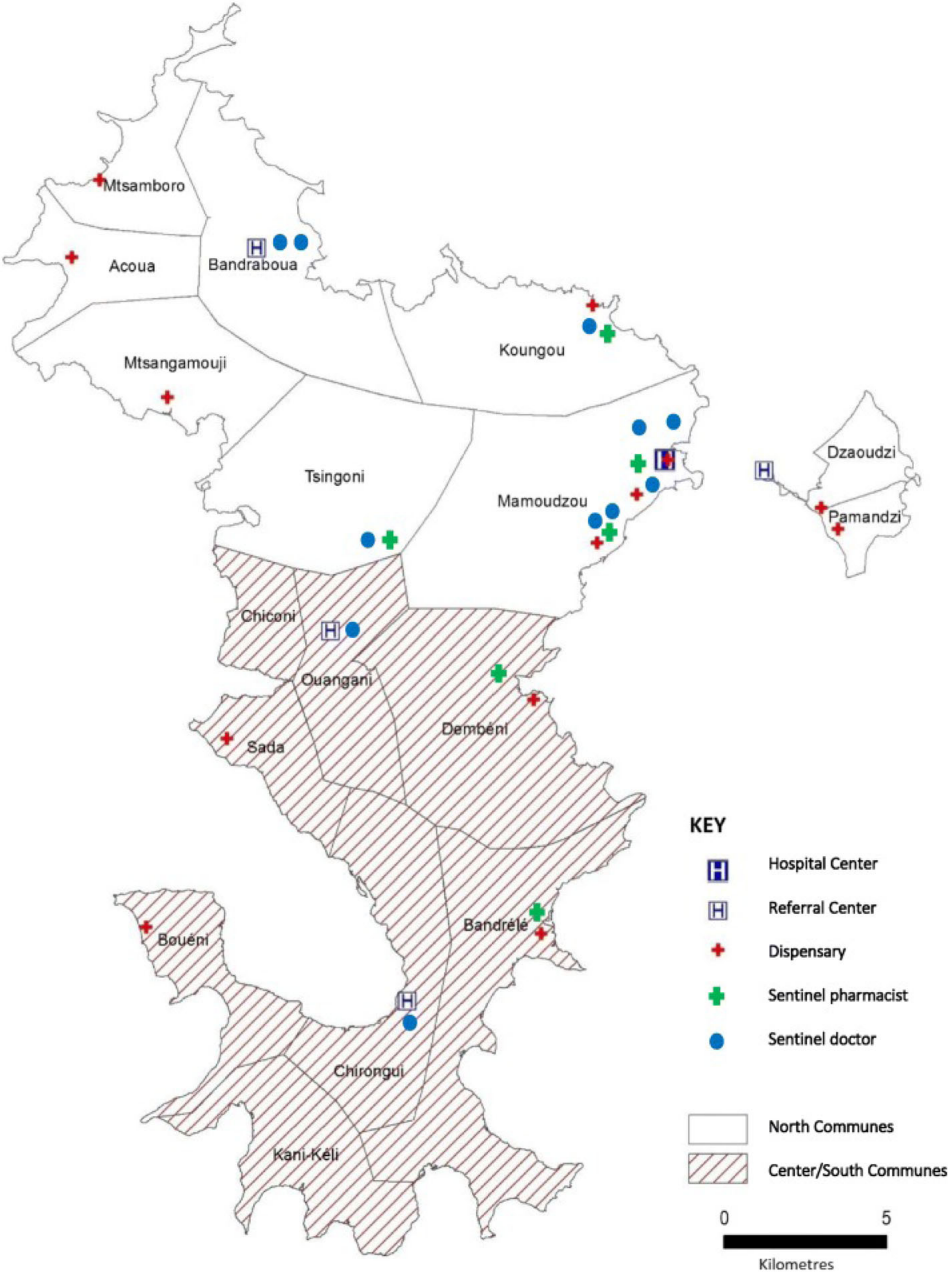
Map of the Mayotte Hospital Center health centers, 2016–2017 [Source: GeoflaIGN, Produced by CIRE OI, 2017]. Map of the communes affected by the water restrictions (center/south and north), the Mayotte Hospital Center health centers, the sentinel pharmacists and doctors, 2016–2017 [Source: GeoflaIGN, Produced by CIRE OI, 2017]

Mayotte is a French territory in the southern hemisphere, between the African continent and Madagascar and in the middle of the Indian Ocean. Its proximity to the Comoros, less than 70 km away, allows a massive immigration from the other islands of the archipelago to that country [19]. The available care consists of a hospital center located in Mamoudzou, the capital of the territory, 4 referral centers and 13 dispensaries (Fig. 1). Altogether these facilities provide 0.8 beds per 1000 inhabitants (2.1 in mainland France). The medical density is 41 per 100,000 inhabitants (156 in mainland France). For pediatrics, medical density is 10 per 100,000 in Mayotte, versus 64 in mainland France. With an incidence of 1/633, SCD is a major public health problem in Mayotte, and because of its social ramifications, it is also a significant social problem in this French overseas territory.

### Study design

This retrospective cohort study was performed using data collected from the medicalized information system program (MISP) of the Center for SCD of the Mamouzou General Hospital in Mayotte. The clinical and biological data collected for this project followed the recommended standard of care of SCD, by the French authority (Haute Autorité de Santé).

### Patients

Patients with SCA or S/beta-thalassemia, younger than 18 years in 2017, were seen every 3 months (with a consultation by a pediatrician specialized in SCD and a standard biological assessment). A specific appointment was scheduled for annual review (during which the TCDwas carried out). These patients were prospectively included in the database between 2007 and 2017, after obtaining a statement of patient’s non opposition, as required by French regulations. For 50% of them, SCD has been diagnosed by universal neonatal screening [15]. The other 50%, born outside France, were diagnosed late in the presence of VOC or other complications of SCD.

### SCD clinical history

Clinical and biological data, collected between January1^st^ 2007 and December 31^st ^2017, were considered for the analysis. The patients were in a stable state when these biological data were taken. For each patient, the following data were collected: age, gender, hemoglobin type, alpha and beta globin genotype, beta globin haplotype, basal HbF level, basal Hb level, glucose-6-phosphate-dehydrogenase (G6PD) status, UGT1A1 gene mutations status, single nucleotide polymorphism (SNP). SNP was genotyped using Single-Tube Fluorescent Bidirectional polymerase chain reaction (PCR). The other variables included severity and number of prior acute or chronic sickle cell specific complications (acute splenic or hepatic sequestration, acute chest syndrome, sickling related painful vasoocclusive crisis (VOC), neurologic events, severe infections, acute anemia, cholelithiasis), use of opioids for painful events, hydroxyurea treatment, number of RBCs transfusions (or RBCs exchange), and number of hospitalizations. These data, from the computerized medical record, were transferred in 2017 to an anonymized database for analysis.

### Definitions

The VOC is apainful complication of SCD [20]. We only collected painful events that required hospital treatment. Hemolytic crisis: decreases in the concentration of hemoglobin (Hb) and hematocrit [21]. Hand-foot syndrome: swelling in the hands and feet with pain and/or local heat, which may also be associated with a decrease in Hb concentration [22]. Infection: fever accompanied by prostration and leukocytosis, with or without other laboratory tests and imaging [21]. Acute splenic sequestration was defined as a sudden increase in the spleen size associated with pain in the left upper quadrant, a decrease in the hemoglobin concentration of at least 2 g/dL and in thrombocytes number [22]. Acute hepatic sequestration was defined as a sudden increase in liver size associated with pain in the right upper quadrant, a decrease in the hemoglobin concentration of at least 2 g/dL, and more abnormal results of liver-function tests not due to biliary tract disease [23]. Acute chest syndrome (ACS) and painful vasoocclusive crisis were defined as previously published [24]. The cerebral vasculopathy results in stroke and subclinical or paucisymptomatic ischemic lesions. It was detected using TCD ultrasonography and magnetic resonance imaging (MRI) [25, 26].

### Exclusion criteria

Were excluded from this study infants under 1 year of age on December31^st^2017, because of their high HbF level. Children lost tofollow-up for more than 3 years were also excluded.

### Statistical analysis

The database was anonymised before analysis. The Statistical Package for the Social Sciences (SPSS) statistical software, version 13.0 (SPSS, Chicago, IL) was used for statistical analysis. The data were described as number and percentages for categorical variables and mean ± standard deviation (SD) or median (range) for continuous variables. Independent Student’st- test was used to compare continuous variables between groups (Kruskal-Wallis test for comparing more than 2 groups), andchi-square test (or Fisher exact test) for categorical data. Multivariable logistic regression was used to examine the association between each of the variables and the sickle subphenotype with adjustment for age and sex. *P* values < 0·05 were considered statistically significant. All acute clinical events, correctlyrecorded in the medical files from birth (or the beginning of follow-up) to the date of the final evaluation were included in the analyses. Kaplan-Meyer curves and log-rank test were performed for generating survey curves. We performed a ROC curve for HbF, which allows to distinguish two groups (HbF < 10% versus HbF > 10%).

### Regulatory and ethical authorizations

All patients or legal representatives (for the children included in the study) gavewritten informed consent to participate in this research. The study cohort was approved by the Mamoudzou Hospital Ethical committeeand the database was declared at the Commission NationaleInformatiqueetLibertés (CNIL N° 2,004,054–11/26/2016).

## Results

**Fig. 2:**
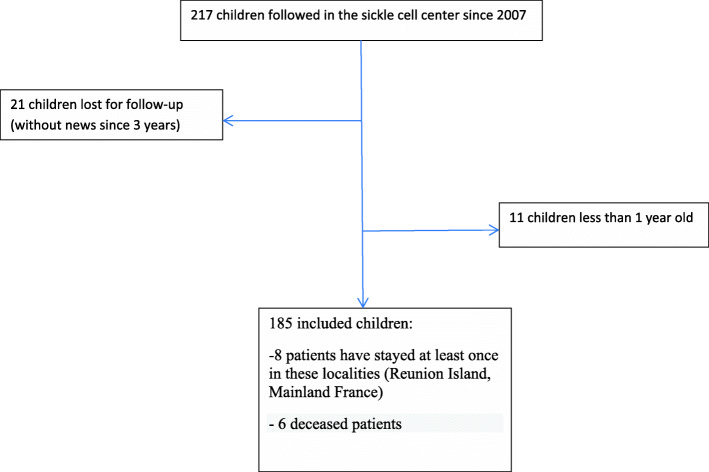
Flow chart describing how the cohort was identified

Ten percent of patients from the Center were lost to follow up (Fig. 2). One hundred and eighty five children were enrolled in this study, 72% with SCA, 16% with Hb/Sβ^0^-thalassemia and 12% with Hb/Sβ+ thalassemia. The mean age was 9.5 years, with ranges from 19 months to 18 years. 15.3% of the children met the definition criteria of cerebral vasculopathy. There were missing data for 22 of included patients.

### Sickle cell genotypes

In our study, homozygous sickle cell patients had significantly lower mean hemoglobin and hematocrit levels than Sβ0 and then Sβ + patients. On the clinical level, SCA was significantly associated with ACS, bacterial infections, cholelithiasis, hospitalizations and more frequent transfusions (Table 1).

**Table 1:** Comparison of patients characteristics according to the sickle genotypes

	SS (*n* = 118)	*Sbeta°* (*n* = 26)	*Sbeta*^*+*^ (*n* = 19)	*P*
	Mean (±SD) or n (%)			
Hb (g/dL)	7,8 (±0,1)	8,3 (±0,3)	8,8 (±0,1)	
Hematocrit (%)	24,1 (±0,3)	27,0 (±0,7)	28,3 (±1,0)	
MCV (fL)	80,3 (±0,9)	65,8 (±1,3)	69,3 (±2,2)	
MCHC (g/dL)	32,7 (±0,1)	31,0 (±0,3)	31,7 (±0,3)	
Reticulocytes (G/L)	268,1 (±9,8)	250,0 (±20,9)	179,1 (±19,0)	0,003
Leukocytes (G/L)	13,2 (±0,4)	10,6 (±0,7)	11,0 (±0,7)	0,007
Hospitalization/year				0,003
*No*	13 (11,1)	8 (32)	7 (38,9)	
*1 to 2*	91 (77,8)	17 (68)	11 (61,1)	
*≥ 3*	13 (11,1)	0	0	
Red Blood Cell Transfusion érythrocytaire				0,001
*Never*	20 (17,1)	13 (52)	8 (42,1)	
*Occasionnally*	78 (66,7)	11 (44)	11 (57,9)	
*Transfusion therapy*	19 (16,2)	1 (4)	0	
Infection	61 (55)	8 (30,8)	6 (31,6)	0,026
Acute Chest Syndrome	27 (23,9)	0	4 (21,1)	0,009
Number of ACS/year				0,041
*Never*	80 (74,1)	25 (100)	15 (78,9)	
*1*	22 (20,4)	0	3 (15,8)	
*≥ 2*	6 (5,5)	0	1 (5,3)	
Cholelithiasis	28 (26,4)	4 (18,2)	0	0,038

### Sickle cell haplotypes

Having at least one Bantu allele concerned almost all of our study population. The patients with Bantu / Bantu haplotype had significantly lower hematocrit, higher MCV and MCHC. They were hospitalized andtransfusedmore often (Table 2).

**Table 2:** Comparison of patients characteristics according to the sickle cell haplotypes

	Homozygous Bantu			Heterozygous Bantu			Homozygous Benin			Heterozygous Benin		
	Bantu/Bantu (*n* = 95)		*P*	Bantu/− (*n* = 46)		*P*	Benin/Benin (*n* = 4)		*P*	Benin/− (*n* = 35)		*p*
	Mean (±SD) or n (%)			Mean (±SD) or n (%)			Mean (±SD) or n (%)			Mean (±SD) or n (%)		
Age (years)										7,98 (±0,82)	11,23 (±0,55)	0,002
Hématocrit (%)	24,18 (±0,38)	26,26(±0,51)	0,001							26,26 (±0,6)	24,43 (±0,36)	0,01
MCV (fL)	81,27 (±1)	67,78 (±1,23)	< 0,001							66,72 (±1,42)	80,04 (±0,97)	< 0,001
MCHC (g/dL)	32,75 (±0,13)	31,43 (±0,24)	< 0,001	32,22 (±0,13)	33,72 (±0,74)	0,023	34,25 (±1,04)	32,23 (±0,13)	0,011			
HbF (%)				13,11 (±0,7)	6,08 (±2,3)	0,044	4,01 (±2,7)	13,07 (±0,7)	0,034	12 (±0,75)	15,1 (±1,52)	0,048
HbS (%)	85,9 (±1,1)	79,67 (±1,36)	0,001							78,68 (±1,62)	85,48 (±0,98)	0,001
TCD									0,041			
*Normal*							1 (33,3)	101 (83,4)				
*Limit*							0	10 (8,3)				
*Pathological*							2 (66,7)	10 (8,3)				
Duration of follow-up (months)				87,57 (±5,03)	143,7 (±30,43)	0,032	153 (±37,4)	87,7 (±5)	0,025			
Newborn screening										29 (74,3)	8 (7,3)	0,001
Treatment Iron chelator							2 (50%)	9 (6,3)	0,028			
Hospitalization/year			0,012									0,002
*No*	9 (9,6)	13 (25,5)								12 (30,7)	10 (9,5)	
*1 à 2*	75 (79,8)	37 (72,5)								27 (69,3)	85 (80)	
*≥3*	10 (10,6)	1 (2)								0	11 (10,5)	
RBC transfusion			0,046									
*Never*	18 (18,9)	19 (37,3)										
*Occasionnally*	63 (66,4)	28 (54,9)										
*chronic transfusion*	14 (14,7)	4 (7,8)										

### Alpha thalessemia

Fifty percent of the patients had a alpha-3.7 mutation of at least one alpha gene. The absence of this alpha-thalassemic mutation was significantly associated with cerebral vasculopathy and more frequent RBC transfusions (Table 3).

**Table 3:** Comparison of patients characteristics according to the alpha thalassemia trait

	Alpha thalassemia trait (*n* = 80)	No alpha thalassemia trait (*n* = 52)	*P*
	Mean (±SD) or n (%)		
Hb (g/d L)	8,2 (±0,1)	7,7 (±0,2)	0,007
Hématocrit (%)	25,6 (±0,4)	23,8 (±0,5)	0,004
MCV (fL)	73,3 (±1)	81,8 (±1,5)	< 0,001
Reticulocytes (G/L)	239,6 (±10)	288,2 (±13,8)	0,004
TCD			0,017
*Normal*	72 (90)	37 (71,2)	
*Limit*	3 (3,7)	8 (15,4)	
*Pathological*	5 (6,3)	7 (13,4)	
Splenomegaly ratio	0,54 (±0,03)	0,43 (±0,05)	0,057
Cerebral vasculopathy			0,004
*Yes*	78 (91,8)	43 (72,9)	
*Pathological TCD/MRI*	7 (8,2)	13 (22)	
*Stroke*	0	3 (5,1)	
RBC Transfusion			0,028
*No*	28 (29,4)	10 (15,6)	
*Occasionnally*	60 (63,2)	42 (65,6)	
*Exchange transfusion*	7 (7,4)	12 (18,8)	

### Single nucleotide polymorphism

Table 4 shows the different SNP associated with the hemolytic subphenotype. The table of patient characteristics according to the SNP, being very complex given the large number of variables, we found it simpler here to describe the data. The presence of Xmn1 in our cohort was significantly associated with higher hemoglobin and hematocrit levels, decreased leukocytes, and a higher splenic ratio. Having two favourable SNP alleles rs4671393 was significantly associated with higher hemoglobin and hematocrit, and a higher HbF for patients under HU treatment, as well as lower HbS. Patients with at least one favourable rs11886868 allele had higher hemoglobin and hematocrit. Patients with at least one favorable rs1427407or rs9399137 alleles had higher HbFlevel. The favourable rs10189857 allele was associated with a low hemoglobin and hematocrit and high leucocytes. Patients with the favourable rs28384513 allele were more frequently diagnosed with the neonatal screening test. The absence of TAC deletion at SNP rs66650371 was significantly associated with higher mortality.

**Table 4:** SNP associated with the hemolytic subphenotype

SNP	Avantageous allele/Disadvantageous allele	Allele frequency (%)	OR (95%CI)	***P***
BCL11A rs4671393	**A**/G	37%	3,13 [1,1-8,89]	**0,047**
BCL11A rs11886868	**C**/T	43%	4,28 [1,6-11,5]	**0,005**
BCL11A rs1427407	**T**/G	15%	4,02 [1,75-9,22]	**0,001**
HMIP rs9399137	**C**/T	7%	5,92 [1,28-27,4]	**0,012**
Xmn1 rs7842144	**T**/C	6%	–	0,76
BCL11A rs10189857	**A**/G	54%	–	0,85
HMIP rs28384513	**C**/A	67%	–	1
HMIP rs66650371	**Deletion**/ACT	37%	–	0,39

### UGT1A1 gene mutations status

The low number of patients with the UGTA1 mutation (*n* = 23, 12%) did not allow statistical analysis.

### Hemoglobin F (Table 5)

**Fig. 3:**
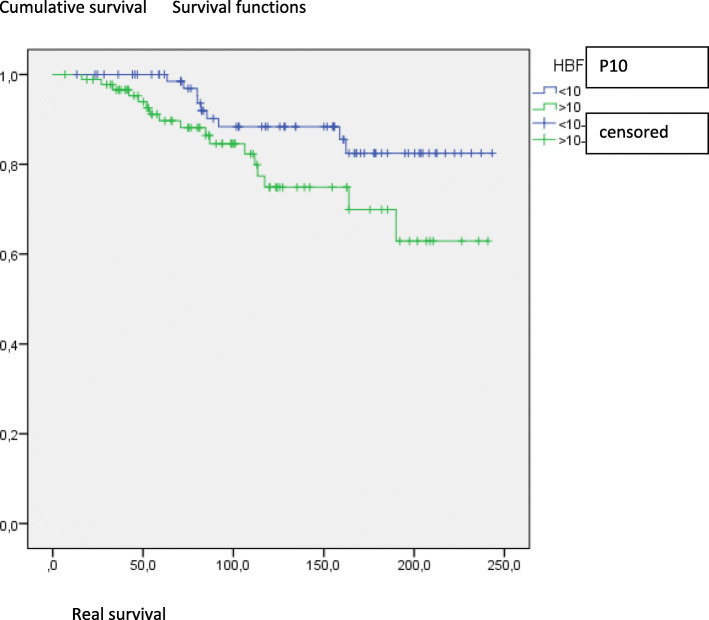
Survival without cerebral vasculopathy according to the Hb F level

The survival analysis without occurrence of cerebral vasculopathy showed that the group of patients with HbF > 10% presented a significantly greater risk of early onset of cerebral vasculopathy, the main complication of the hemolytic sub-phenotype (Fig. 3). The group with low HbF was associated with vaso-occlusive complications. Homozygous Bantu patients in the HbF group> 10% were was associated with an increase in hemoglobin level in less hospitalized (*p* = 0.002), less transfused (*p* = 0.025), had less VOC / year (*p* = 0.039), but they had more cerebral vasculopathy (*p* = 0.023) than those with < 10% HbF. Homozygous Bantu patients in the HbF group < 10% had less cholelithiasis (*p* = 0.021). Patients in both groups, when they carried one or two Benin haplotypes, were less hospitalized (*p* = 0.002), had less VOC per year (*p* = 0.039) and their 1st VOC occurred less early (*p* = 0.03) than those that did not have any Benin haplotypes. Only the patients heterozygous for Benin haplotypes had a significant high HbF level (*p* = 0.04). Patients who do not carry a Benin allele were more transfused (*p* = 0.018) than those who did. The alpha-thalassemic mutation was associated with an increase in hemoglobin level in patients at risk of vasculopathy (*p* = 0.023), and an increased leukocyte rate (*p* = 0.001). Children in the group with an alpha mutation were hospitalized less often (*p* = 0.004) and were less likely to have cholelithiasis (*p* = 0.041) than other children. Children in the < 10% HbF group who carried an alpha mutation received fewer transfusions than those > 10% (*p* = 0.048). Multivariate analysis (Table 5) did not find any independent genotypic marker. However, some SNPs were close to significance: BCL11A rs1427407 (*p* = 0.051) and BCL11A rs11886868 (*p* = 0.06). BCL11A rs4671393 (*p* = 0.2) and HMIP rs9399137 (*p* = 0.24) were not independently associated with the phenotypic groups. A concordance chi-2 test found preferential associations between some SNPs (Table 6). The linkage imbalance between BCL11A rs66650371 and rs9399137 was highly significant for a large number.

**Table 5:** Characteristics of the patients followed in Mayotte according to the HbF level

Profile	HbF ≥ 10%	HbF < 10%	OR (95%CI)	*P*	P*
**Age, Mean (SD)**	8,6 (±0,5)	12,1 (±0,6)		< 0,001	0.09
**Hemoglobin sickle cell genotype n (%)**				0.187	0.2
*HbSS*	58 (68,2)	60 (76,9)			
*HbS/β°Thalassemia*	18 (21,2)	8 (10,3)			
*HbS/β*^*+*^*Thalassemia*	9 (10,5)	10 (12,8)			
**Haplotypes n (%)**					
*Bantu/−*	79 (97,5)	62 (93,9)		0.41	0.45
*Bantu/Bantu*	48 (59,3)	47 (71,2)		0.14	0.18
*Benin/−*	27 (69,2)	12 (18,2)	2,25 [1,03-4,9]	0.04	0.4
*Benin/Benin*	1 (1,2)	3 (4,5)		0.33	0.5
**Alpha thalassemia n(%)**	56 (60,2)	42 (58,3)		0.87	0.8
G6PD deficiency n(%)					
*G6PD-Deficiency*	5 (6,4)	7 (10,3)		0.02	0.2
*Heterozygote*	4 (5,1)	12 (17,6)			
**UGT1A1 mutation n (%)**	12 (14)	11 (15,1)		1	1
**SNP n (%)**					
*BCL11A or rs4671393*	20 (19,8)	7 (8)	3,13 [1,1-8,89}	0.047	0.2
*BCL11A rs11886868*	28 (63,6)	9 (29)	4,28 [1,6-11,5]	0.005	**0.06**
*BCL11A rs1427407*	28 (41,8)	10 (15,2)	4 [1,75-9,22]	0.001	**0.051**
*HMIP rs9399137*	12 (14,3)	2 (2,7)	5,92 [1,28-27,4]	0.01	0.24
*Xmn1 or rs7842144*	6 (7,2)	4 (5,7)		0.76	0.74
*BCL11A rs10189857*	64 (79)	55 (77,5)		0.85	0.8
*HMIP rs28384513*	30 (73,2)	23 (74,2)		1	1
*HMIP rs66650371*	31 (41,3)	22 (33,3)		0.39	0.4
*HMIP rs4895441*	7 (17,1)	2 (6,5)		0.28	0.3
**Hydroxyurea treatment**	11 (11)	19 (22,1)		0.047	0.5
**Osteonecrosis (n, %)**	1 (1,3)	8 (11,1)			
**Number of hospitalization (n, %)**				0.01	0.1
*No*	21 (21)	14 (16,5)			
*1 à 2 per year*	77 (77)	60 (70,6)			
*≥ 3 per year*	2 (2)	11 (12,9)			

P* obtained after a multivariate analysis

**Table 6:** Linkage imbalances between SNPs according to the number of studied samples

Locus	Single nucleotide polymorphism	N	Concordance (*p*)
BCL11A	rs11886868-rs1427407	54	< 0.001
	rs1427407-rs4671319	51	< 0.001
HMIP	rs9399137-rs4895441	72	< 0.001
	rs66650371-rs9399137	141	< 0.001

### G6PD deficiency

Patients with G6PD mutation had a greater MCV (*p* = 0.05), and more infections (*p* = 0.045) than those without. Regarding patient management, TCD was performed more often (*p* = 0.026), iron chelatorsand transfusion were prescribed more often (*p* = 0.001and *p* = 0.045, respectively).

## Discussion

According to our working hypothesis, the HbF level could direct us towards a sub-phenotype of the disease. We therefore looked for a HbF value to determine these two sub-phenotypes. Our study population was characterized by the predominance of sickle cell anemia, with a severe clinical presentation [27], followed by the compound heterozygous HbS/βthalassemia. The Bantu haplotype, accounting for 80% of the alleles, reflects the East African origin of the Mahoran population [28]. Compared to the previous study conducted in Mayotte, haplotypes seemed to diversify: 64.9% of homozygous Bantou in 2017, against 88% in 2011 [18]. The Benin, Cameroon and Senegal haplotypes appeared or became more frequent in the past 6 years. Intense immigration to Mayotte could partly explain this result. But, this should be taken with caution even if the inclusion criteria were not the same, the previous study only considering children who had been diagnosed by the neonatal screening. The Bantu haplotype was not directly related to a particular phenotypic group, but increased the risk of cerebral vasculopathy in patients with HbF > 10%. It was probably difficult to highlight a statistical link because of its very high frequency in our population. The Bantu haplotype is classically associated with a more severe prognosis, and appears to be related to greater hemolysis in a study comparing Jamaican and Ugandan populations, and in another involving a Brazilian cohort [29, 30]. The Benin haplotype was associated with the vasooclusive phenotype in our study. It corresponded to more severe phenotypes than other haplotypes (Senegal, Arabo-Indian), but is not known to be associated with the risk of cerebral vasculopathy. The G6PD mutation was associated with more transfusions because of lower Hb levels. This link was not found at the level of phenotypic groups. The studies on this subject obtained different results: G6PD deficiency leads to a hemolytic phenotype according to some French studies [31, 32], and does not affect this phenotype according to others [33–37]. Our study investigated three mutations, but did not collect the molecular and clinical expression of G6PD deficiency. It didnot take into account the possible presence of other mutations, and possible chromosomal inactivation by lyonization. It would be interesting to specifythe residual enzymatic activity and the clinical complications presented by the patients.

### HbF is associated with a high risk of cerebral vasculopathy

Our survival analysis without occurrence of cerebral vasculopathy showed that the group of patients with HbF > 10% presented a significant risk of early onset of cerebral vasculopathy. Even if predicting sickle cell severity is complex, stroke appears to be the most devastating complication of sickle cell anemia (SCA), affecting up to 30% of children with the disease. Despite the relative frequency of stroke in SCA, few predictors of this risk have been described [38–40]. Thus our severity classification based on the “existence or not of the risk of cerebral vasculopathy” enabled us to better characterize the role of genetic modifiers of SCA. By inhibiting HbS polymerization and reducing the tissue injury, HbF is the predominant modulator of the phenotype of sickle cell anemia [40]. Our patients with high hemoglobin F had less VOC, and were hospitalizedless often. Because of their less preoccupying symptomatology, they were less often seen in the follow-up consultation. As a result, they were at greater risk of developing silent cerebral vasculopathy, with diagnostic delays since they did not benefit from regular DTC. On the contrary, low HbF was associated with vaso-occlusive complications, requiring treatment with hydroxycarbamide (HU). However hydroxycarbamide is the only HbF inducer approved for the treatment of SCD [39]. As reported in several studies, HbF levels have a clinically beneficial effect on SCD [40]. Bantu and Benin haplotypes also express relatively lower Hb F levels, with a severe clinical presentation. Indeed, among the predictors of survival, HbF levels play a significant role in lowering the morbidity and mortality. Co-inheritance of HbS and hereditary persistence of fetal hemoglobin (HPFH) may contribute to variable HbF levels in SCD patients, thus influencing their clinicopathological profile [40]. In fact, in patients with HbF > 10%, there were observed a residual risk of vasculopathy when risk of VOC disappears. It is known that in SCD patient recurrent stroke persists until HbS decreases to 30%, needing high level of HbF in patients without blood transfusion [41]. HbF inhibits HbS polymerization and its abundance in the red blood cells dilutes down the concentration of HbS. In 2012, Steinberg et al. synthesized the results of studies on the association between HbF and sickle cell clinical phenotype. They found no or little evidence of a protective effect of HbF on cerebral vasculopathy, pulmonary arterial hypertension, priapism and glomerulopathy [15]. Indeed, α-thalassemia has been shown to diminish the severity of disease by reducing the amount of sickled RBC, decreasing the intracellular HbS level, and also increasing HbF level. Our study showed a high prevalence of 3.7 kb α-globin gene deletion. This has also been reported among SCA patients in Tanzania [42], in Guadeloupe [43], in Brazil [44], in India [45], in Saudi Arabia [46], in France among Africans [7], and in Cameroon [47]. The beneficial effect of HbF is explained by its ability to prevent sickling. However, the intra-erythrocyte distribution of HbF is heterogeneous. Also, BCL11A and HBS1L-MYB SNPs in the β-globin gene have been found to be associated witha high level of HbF, usually under conditions of poor erythropoiesis, such as SCD [7].

### Correlation of genotype to subphenotypes

#### SNPs associated with high Hb F level

Investigation of genetic variants has identified several genes as principal influencers of HbF regulation. In our study, the alleles BCL11A rs1427407, HMIP rs4895441 and HMIP rs9399137 were significantly associated with an increase in HbF. In the literature, these SNPs are indeed strongly associated with HbF. BCL11A rs1427407 was the SNP with the highest correlation withHbF in a Genome wide association study (GWAS) performed in Tanzania [48]. SNPs BCL11A rs4671393, BCL11A rs11886868, and HMIP rs4895441 increase the induction of HbF with hydroxycarbamide. This effect was found in several cohorts (North America, Brazil), where BCL11A was most strongly associated with an increase in HbF under hydroxycarbamide, regardless of its effect on basal HbF [7, 49]. The mechanism of action is not explained. The association of SNPs with HbF varies between populations of different origins, so some SNPs have no effect in some populations. This was the case forXmn1 in our cohort, which may have resulted from its rarity. A study comparing two cohorts of European and African origin observed differences in allele frequency and correlation with HbF [50]. Another study, conducted in Cameroon, showed identic allelic frequencies between a Cameroonian population and the African-American cohort, but a lower impact on HbF among Africans [51]. These results show the interest of looking for SNPs in a given population by performing GWAS, and not simply extrapolating the polymorphisms found in another population. The African continent in particular could benefit from more GWAS polymorphisms Xmn1. BCL11A rs4671393 and BCL11A rs11886868 are associated with elevated hemoglobin. This result is found in other African studies [48, 52]. HMIP rs66650371 is correlated with a decrease in mortality on a small population in our cohort, which is not reported (to our knowledge) in the literature.

The most remarkable result of our study was the association of SNPs with the phenotypic groups that we aimed to determine. BCL11A rs4671393, BCL11A rs11886868, BCL11A rs1427407 and HMIP rs9399137 were correlated with the HbF group> 10%, which presents a higher risk of cerebral vasculopathy and would be oriented towards the hemolytic sub-phenotype. BCL11A rs1427407 was the most strongly associated in our population, which corresponds to its strong correlation with HbF found in the Tanzanian GWAS [48]. HMIP rs9399137 is the HMIP polymorphism most strongly associated with HbF levels in African populations [53]. Multivariate analysis found no independent association of these SNPs with clinical profiles, BCL11A rs1427407 being close to significance. There are therefore unknown factors (interactions, intermediate factors, or other SNPs in linkage disequilibrium) that intervene in this genotype-phenotype correlation HMIP rs66650371 was not associated with either HbF or a phenotypic group in our cohort. This deletion of 3 bases, in linkage disequilibrium with rs9399137 in the literature as in our study, is located at the binding sites of four essential transcription factors in erythroid differentiation. It inhibits the expression of MYB, and thus leads to both an acceleration of differentiation (responsible for an increase in HbF) and a decrease in erythrocyte proliferation (which could cause a decrease in hemoglobin) [7, 53, 54]. These two effects could explain the lack of correlation with the clinical phenotype. The favorable SNP rs66650371 is less common in African populations and particularly in our cohort, which may also explain the lack of observed link. We also did not find any clinical phenotypic association for the SNP Xmn1, which is also infrequent in our population. This geno-phenotypic clinical association in sickle cell disease is interesting because it is poorly described in the literature. In 2008, Lettre found a significant link between the association of 5 SNPs (BCL11A rs4671393, HMIP rs28384513, rs9399137 and rs4895441, and XmnI rs7482144) and the reduction of VOCs in the SCD cohort [55]. These SNPs are also associated with a less severe clinical phenotype in another pathology of hemoglobin, beta-thalassemia [56]. The results of Lettre and other studies show a stronger geno-phenotypic correlation when several SNPs are associated [55, 57]. It would therefore be interesting to study the link between these sets of specific polymorphisms and the sub-phenotypes of sickle cell disease. Our study found an association between some SNPs and the risk of cerebral vasculopathy; this link depends on the frequency of the polymorphism, the correlation rate according to the population, and could be amplified by the association of these SNPs.

### The alpha-thalassemic mutation is a vaso-occlusive profile

The alpha-thalassemic mutation was associated with a decrease of hemolysis biological parameters (anemia, reticulocytes), and less cerebral vasculopathy. In the literature, it is also associated with fewer vascular complications [15, 58, 59]. This mutation decreases the parameters and complications of hemolysis in the at-risk group of vasculopathy. It protects against vascular complications, even in patients who are at high risk. This is due to the decrease in HbS concentration in erythrocytes, which leads to a decrease in hemolysis [31, 60]. The resulting increase in blood viscosity favors vaso-occlusive complications [60].

### Limitations and interests of our study

Our determination of the sickle cell sub-phenotypes from the HbF level didnot yield the expected result, although some trends have emerged. Difficulties in monitoring the Mahoran pediatric patients lead to poor control of environmental prognostic factors such as lifestyle, therapeutic education of the patient, screening and early management, and regular monitoring. This may have impacted some results of our study. However, we relied on the fact that environmental factors do not appear to affect the type of expression of the disease [18]. The number of missing data, which is too high for some parameters, requires further study. The analyzed SNPs were not, for some, the most frequent or the most strongly associated with the HbF level in an African population. GWAS and genotype-phenotype correlation research must be adapted to different types of populations for a better global understanding of SCD. Our results, need to be further developed, could make it possible to predict early (in utero or during the neonatal period) the type of complication that the sickle cell child will present, and thus to predict the type of surveillance and treatment required for each patient. They could help in the decision of intensiveinterventionssuch as bone marrow transplantations.

## Conclusion

Our study allowed a description of the Mahoran pediatric population, reflecting the need to continue to improve monitoring clinical data continuously. In our cohort, the SNPs BCL11A rs4671393, BCL11A rs11886868, BCL11A rs1427407 and HMIP rs9399137 were associated with the group of children with HbF > 10%, and which seemed to present a high risk of occurrence of cerebral vasculopathy. This link was not found independently for each SNP. Beta-globin haplotypes and alpha-thalassemic mutations might also influence the clinical expression of the disease, but the multivariate analysis did not find any independent genotypic marker.

## Data Availability

Our database is available from the corresponding author on reasonable request.

## Abbreviations

SCD: Sickle cell disease
WBC: White blood cells
RBC: Red blood cells
HbF: Fetal hemoglobin
G6PD: Glucose-6-phosphate-dehydrogenase
UGT1A1: Uridine-diphosphoglucuronate glucuronosyltransferase
VOC: Vasoocclusive crisis
Hb: Hemoglobin
ACS: Acute chest syndrome
SD: Standard deviation
SCA: Sickle cell anemia
MCV: Mean Corpuscular Volume
MCHC: Mean Corpuscular Hemoglobin Concentration
SNP: Single nucleotide polymorphism
HU: Hydroxy-urea
GWAS: Genome wide association study
